# The Instrument Design of Lightweight and Large Field of View High-Resolution Hyperspectral Camera

**DOI:** 10.3390/s21072276

**Published:** 2021-03-24

**Authors:** Xinghao Fan, Chunyu Liu, Shuai Liu, Yunqiang Xie, Liangliang Zheng, Tiancong Wang, Qinping Feng

**Affiliations:** 1 Changchun Institute of Optics, Fine Mechanics and Physics, Chinese Academy of Sciences, Changchun 130033, China; xinghao.fan@foxmail.com (X.F.); liushuai@ciomp.ac.cn (S.L.); xieyunqiang131@163.com (Y.X.); adqe@163.com (L.Z.); wtcshuaige@sina.com (T.W.); victor.2008.happy@163.com (Q.F.); 2 University of Chinese Academy of Sciences, Beijing 100049, China; 3 Key Laboratory of Space-Based Dynamic & Rapid Optical Imaging Technology, Chinese Academy of Sciences, Changchun 130033, China

**Keywords:** linear variable filter, digital domain TDI, high ground resolution, hyperspectral, camera

## Abstract

The design of compact hyperspectral cameras with high ground resolution and large field of view (FOV) is a challenging problem in the field of remote sensing. In this paper, the time-delayed integration (TDI) of the digital domain is applied to solve the issue of insufficient light energy brought by high spatial resolution, and a hyperspectral camera with linear variable filters suitable for digital domain TDI technology is further designed. The camera has a wavelength range of 450–950 nm, with an average spectral resolution of 10.2 nm. The paper also analyzed the effects of digital domain TDI on the signal–noise ratio (SNR) and the spectral resolution. During its working in orbits, we have obtained high-SNR images with a swath width of 150 km, and a ground sample distance (GSD) of 10 m @ 500 km. The design of the hyperspectral camera has an improved spatial resolution while reducing the cost.

## 1. Introduction

Hyperspectral remote sensing is a technology used to achieve the integrated observation of spectral, spatial, and radiometric information [1,2,3], which is widely applied in the fields of atmospheric detection, Earth resource census, military reconnaissance, environmental monitoring, agriculture, and marine remote sensing [4,5,6].

Benefiting from the low cost of microsatellite platforms, as well as its short development cycles and rapidly evolving technologies, scholars from all over the world are attracted to design compact hyperspectral cameras in order to improve the ability of Earth observation. Recently, extensive studies are carried out to improve the performance of the hyperspectral camera, such as its weight, as well as field of view, spectral resolution, etc. GOMX-4 [7] and Aalto-1 [8] have a mass of only 1.1 kg and 600 g respectively, but their spatial resolution is no better than 70 m. In the future, studies on lenslet array and metasurface will probably be used in the area of weight reduction [9,10]. In terms of FOV, researchers from the University of Chinese Academy of Science used a compact prism spectrometer to increase the swath width of the hyperspectral camera to 5000 pixels [11,12]. Due to insufficient energy, it is more difficult for miniaturized hyperspectral cameras to improve spatial resolution than to increase the field of view and reduce the mass. The COMIS camera on STSAT3 satellite used a large relative aperture optical system to achieve a spatial resolution of 30 m at an orbit of 700 km [13]. Speed compensation technology is used by researchers from the European Space Agency [14] and Changchun Institute of Optics, Fine Mechanics and Physics of Chinese Academy of Sciences [15] to solve insufficient energy at a high spatial resolution. Among them, CHRIS can obtain 20 m spatial resolution hyperspectral images from a 500 km orbit. However, the speed compensation method has high requirements for satellite attitude control, which increases the design difficulty of small satellites.

Based on this, we used digital domain time-delayed integration (TDI) technology to solve the problem of insufficient energy in hyperspectral imaging at high spatial resolution and designed a hyperspectral camera suitable for digital domain TDI technology with linear variable filter.

It is worth mentioning that the imaging methods, especially those used on the cameras carried by unmanned aerial vehicles, have been broadly explored. Among them, the coded aperture hyperspectral camera, pixel array filter hyperspectral camera, compound eye filter hyperspectral camera, and snapshot hyperspectral camera can obtain two-dimensional spatial information and one-dimensional spectral information simultaneously [16,17]. The hyperspectral camera with the imaging method of sub-sampling can easily replace the components to change the wavelength or collect light-field information [18]. The wavelength of hyperspectral cameras with acousto-optic tunable filter (AOTF) and liquid crystal tunable filter (LCTF) are continuously tunable [16,17]. However, the hyperspectral cameras with AOTF and LCTF are suitable for a stationary scene rather than the push-broom imaging method, which is often used by low-orbit satellites. On the other hand, the satellites are far from the target, making it hard for the cameras on the satellites to improve spatial resolution. Therefore, the linear variable filter is more suitable for the hyperspectral cameras on low-orbit satellites than the imaging methods, which are weak in spatial resolution, such as pixel array filter, coded aperture, and snapshot.

This article introduces the principle of TDI in the digital domain, and shows the design of the optical system and electronic system of the hyperspectral camera. Then, the paper presents the test data of hyperspectral cameras in the laboratory and analyzes the influence of the digital domain TDI of different integral grades on the SNR and the spectral resolution. Finally, the in-orbit images obtained by the hyperspectral camera are shown and analyzed.

## 2. Principle and System Design

**Figure 1:**
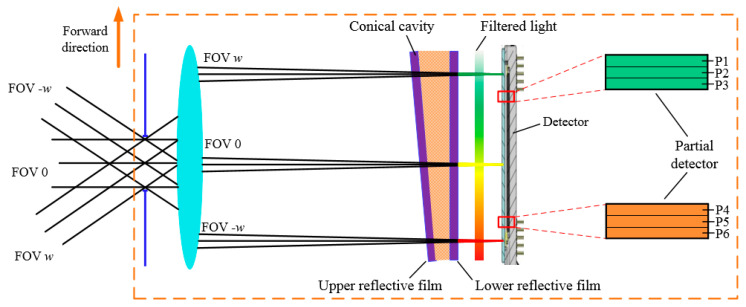
Schematic diagram of filter splitting.

The essence of the digital domain TDI is the addition of multiple image signals. Therefore, it is necessary to image the same object several times during the orbit flight of the camera. In order to cooperate with TDI technology, the camera designed in this paper uses the linear variable filter placed close to the detector to obtain hyperspectral information, as shown in Figure 1. The polychromatic light converges through the telescope and is selected as monochromatic light by the linear variable filter before being imaged on the detector. As a result of the lack of slits, the same object can be captured by different detector row pixels at different times. Another advantage is that each filter is a separate splitter module, making it easy to increase the field of view and miniaturize the instrument.

The wavelength (λ0) at peak transmission of the linear variable filter can be calculated by Formula (1) [19]. According to the formula, the peak transmission wavelength of the filter is mainly affected by the thickness of the resonator. On the one hand, the peak transmission wavelength changes linearly along the variable direction of the resonator thickness. On the other hand, the resonator thickness of adjacent pixel rows has little difference, so the peak transmission wavelength of pixel rows shown as P1–P3 in the Figure 1 has little difference. When TDI integration of P1–P3 is performed as the same band, there should be only a slight spectral broadening.

(1) $$\lambda_{0}=\frac{2nd}{k+(\varphi_{1}+\varphi_{2})/2\pi },$$

(2) $$SNR=20\log\left(\frac{N_{s}}{N_{noise}}\right)=20\log\left(\frac{N_{s}}{\sqrt{N_{s}+N_{r}+N_{d}}}\right),$$

**Figure 2:**
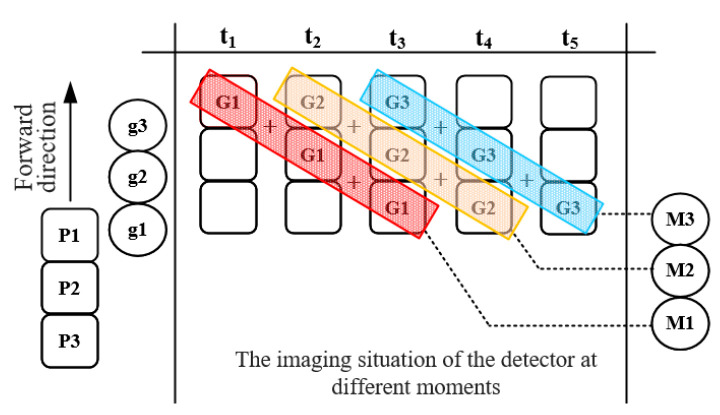
The principle of the digital domain time-delayed integration (TDI).

Digital domain TDI will accumulate the images of the same object acquired by the camera at different moments, as shown in Figure 2. G is the corresponding image of the ground unit “g” on the detector P, and t1-t5 lists the imaging conditions of the detector at different times. As the satellite moves along the orbit, the pixel corresponding to the ground unit “g1” changes from P1 to P3, and “g1” will be imaged as G1 at each moment. The Image G1 includes the read-out noise electrons of the detector (*N_r_*), the dark current noise electrons of the detector (*N_d_*), and the number of photogenerated electrons of the object (*N_s_*). The process of adding up all the image G1 at time t_1_-t_3_ to obtain the image M1 is called a level 3 integral image of g1. According to equation (2), the SNR of image M1 is about 1.73 times that of G1 [20].

We can use traditional Charge-coupled Device (CCD) or Complementary Metal Oxide Semiconductor (CMOS) with the imaging method in Figure 2 to achieve digital domain TDI, but the speed of the satellite relative to the ground is about 7 km/s and the spatial resolution is 10 m, which requires the detector to have a frame rate higher than 700 fps. Therefore, we use the GSENSE5130 detector, which supports setting some pixel rows of the detector as working rows and others as non-working rows to increase the frame rate. When 256 rows of pixels are selected as the working row, the frame rate is better than 1550 fps. The parameters of the detector and the parameters of the camera are shown in Table 1.

### 2.1. Optical System Design

The optical system is used to collect the spatial information, spectral information, and radiation information of the target, directly affecting the image quality and spectral resolution of the camera. The structure of the optical system is shown in Figure 2. The light that contains ground target information is collected by the lens group and condensed on the imaging plane. Detectors with linear variable filter are placed at the imaging plane to convert optical information into electrical information.

**Figure 3:**
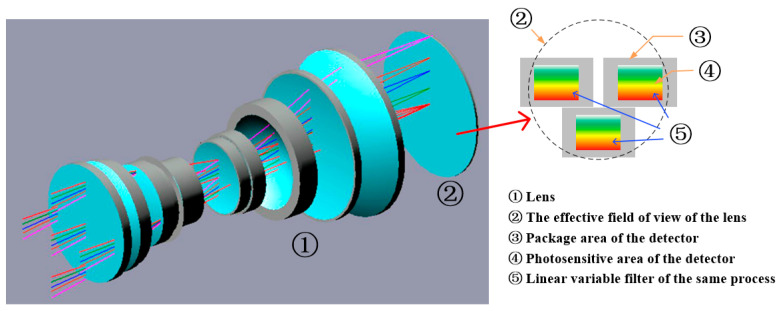
Structure diagram of optical system.

The optical system uses a transmission structure, which can provide a large effective field of view. As shown in Figure 3, the effective field of view of the optical system with a focal length of 213 mm and a field angle of 20.5° is a circle with a radius of 38.5 mm, which can accommodate three detectors placed in a staggered manner. Adjacent detectors overlap 50 pixels horizontally. Through processing, the images obtained by the three detectors can be merged into a large image with line pixels exceeding 15,000.

The linear variable filter as a beam splitting element is embedded in the telescope and does not require the addition of a spectral system like prism and grating imaging spectrometers, so it is very conducive to the weight reduction of hyperspectral cameras [21,22,23]. In actual operation, the protective glass of the detector is replaced with the linear variable filter, a 1 mm thick glass sheet, so that the filter can be placed close to the detector.

**Figure 4:**
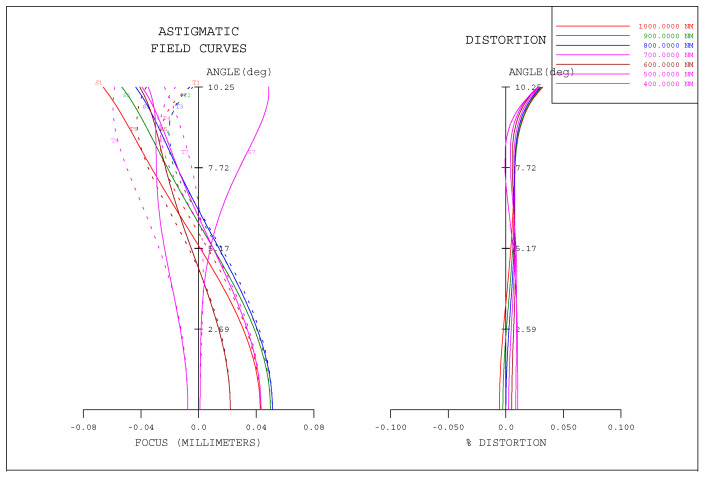
Field curves and distortion curves of optical system.

The optical system is designed specifically for large field of view imaging. The distortion of the optical system in the full field of view is less than 0.035%, and the distortion in the 80% field of view is less than 0.01%, as shown in Figure 4. Therefore, the consistency of ground objects corresponding to adjacent eight lines of pixels in any region of three detectors is greater than 98%, which ensures the accuracy of TDI in the digital domain.

### 2.2. Electronics System Design

**Figure 5:**
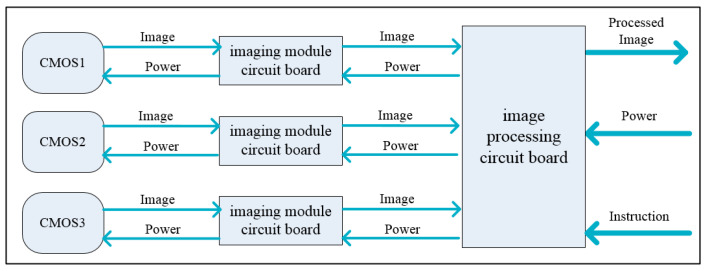
Schematic diagram of electronic system.

The electronics system of the hyperspectral camera is composed of three detectors, three imaging module circuit boards, and an image processing circuit board, among which the detectors use the low-cost and high-performance CMOS sensors. As shown in Figure 5, each of the three CMOS sensors corresponds to an imaging module circuit board. The three imaging module circuit boards are simultaneously connected to an image processing circuit board. The hyperspectral camera exchanges image data and instructions with the satellite by the image processing circuit board.

The image processing circuit board can conduct digital domain time delay integral (TDI) processing for the images acquired by each CMOS. For example, the imaging situation of pixel lines P1–P3 in Figure 2 at the time of t_1_–t_3_ is successively input into the image processing circuit board, and the image processing circuit board will output the integrated image M1 at the time of t_4_. Similarly, M2 and M3 are output at the time of t_5_ and t_6_ respectively. The digital domain TDI process is a “blind box” for the satellite. After receiving the instruction from the satellite, the camera will transmit the processed image to the satellite.

## 3. Laboratory Testing and Evaluation

### 3.1. Camera Calibration

**Figure 6:**
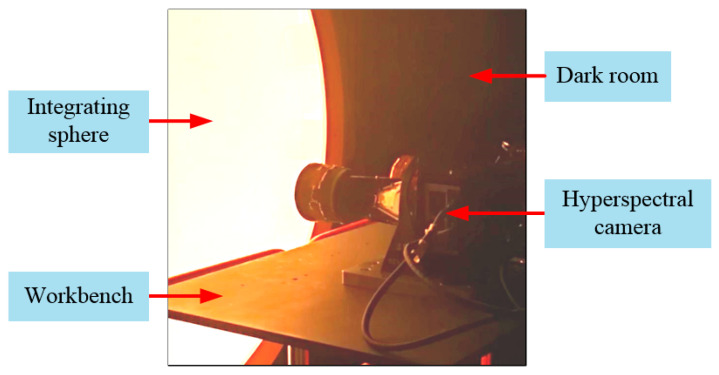
Experimental diagram of radiation calibration.

The hyperspectral camera belongs to a typical radiance observation system. In order to accurately invert the reflectivity of ground objects in each output spectrum, the camera needs to be calibrated for radiation and spectrum in the laboratory after processing and assembly. The experimental device is shown in Figure 6, in which the light emitted by the integrating sphere can fill the aperture and field of view of camera.

**Figure 7:**
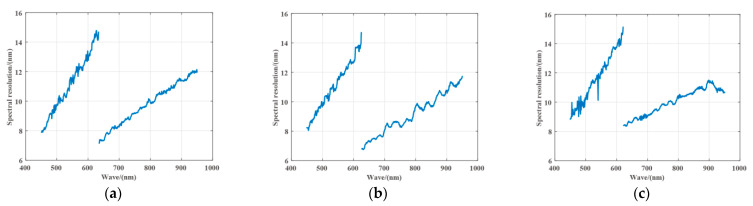
Spectral resolution of the camera. (**a**) CMOS 1; (**b**) CMOS 2; (**c**) CMOS 3.

After processing the experimental data, the spectral resolution of each detector in the 450 nm to 950 nm spectral range is shown in Figure 7. It can be seen from the figure that the spectral resolution of each detector has difference, but the variation trend of spectral resolution is the same. The spectral resolution is about 2% of the center wavelength in the shorter wavelength range (around 450–620 nm), and the spectral resolution is about 1.2% of the center wavelength in the longer wavelength range (around 620–950 nm). The reason for this phenomenon is that the linear variable filter has been specially processed to increase the light energy by increasing the channel bandwidth in the shorter wavelength range so as to improve the SNR in the shorter wavelength range.

The hyperspectral camera has a total of 501 bands, corresponding to the spectrum information of the 450–950 nm with the sampling of 1 nm. However, limited by the data transmission capability, the hyperspectral camera cannot output all spectral information of 450–950 nm. When working on the orbit, any 32 of the 501 bands can be selected as the output spectral bands of the hyperspectral camera according to the needs of the task. In this paper, we selected 32 output bands from 466 nm to 931 nm with a sampling of 15 nm as an example and researched the influence of TDI in the digital domain based on this example.

### 3.2. SNR Calculation

According to Equation (2), the SNR of the image can be calculated by the read-out noise electrons of the detector (Nr), the dark current noise electrons of the detector (Nd), and the number of photogenerated electrons of the object (Ns). Among them, Nr and Nd are determined by the performance of the detector. However, Ns is affected by many factors, such as ground pixel area, ground irradiance, aperture of optical system, and exposure time of detector, etc., as shown in Formula (1) [20].

(3) $$N_{s}=\frac{\rho MS\cos\alpha \cos\beta A\tau \eta \varepsilon t}{n(hc/\lambda )L^{2}},$$

In the formula, *M* is the irradiance of the ground pixel, *A* is the entrance pupil area of the optical system, *S* is the effective reflection area of the space target, *α* and *β* are the angles between the incident direction of sunlight and the direction of light exit and the normal direction of the target surface, and *ρ* is the reflectivity of the target. *L* is the distance between the target and the hyperspectral camera, *τ* is the transmittance of the optical system, *t* is the exposure time, *η* is the quantum efficiency, *n* is the number of pixels occupied by the target spot on the detector, and *ε* is the fill factor of the pixel.

In the 500 km orbit, the speed of the satellite relative to the ground is about 7.2 km/s, so when the ground resolution is 10 m, the exposure time t of the imaging spectrometer can be calculated to be about 1.4 ms. The quantum efficiency, pixel fill factor, read-out noise electrons, and dark current noise electrons can be obtained from the detector manual. And the transmittance of the optical system can be calculated by considering the transmittance of the lens group and the transmittance of the filter. By combining Formulas (2) and (3), the SNR of each output band can be calculated. In the following Table 2, we take CMOS1 as an example and give the SNR calculation results of B1, B7, B13, B20, B27, and B32 in CMOS1 under the illumination condition of 30° sun altitude and 0.05.

It can be seen that the SNR of the single imaging of the system is very low, especially the SNR at B32 affected by the low quantum efficiency of the detector at less than 20 dB. With the increase of TDI levels, the SNR of all spectral bands increases significantly. When using level 8 TDI, the SNR near B32 increases to 28.86 dB, while the SNR of other spectral bands is greater than 30 dB, which can meet the application requirements.

### 3.3. The Effect of TDI on Spectral Resolution

**Figure 8:**
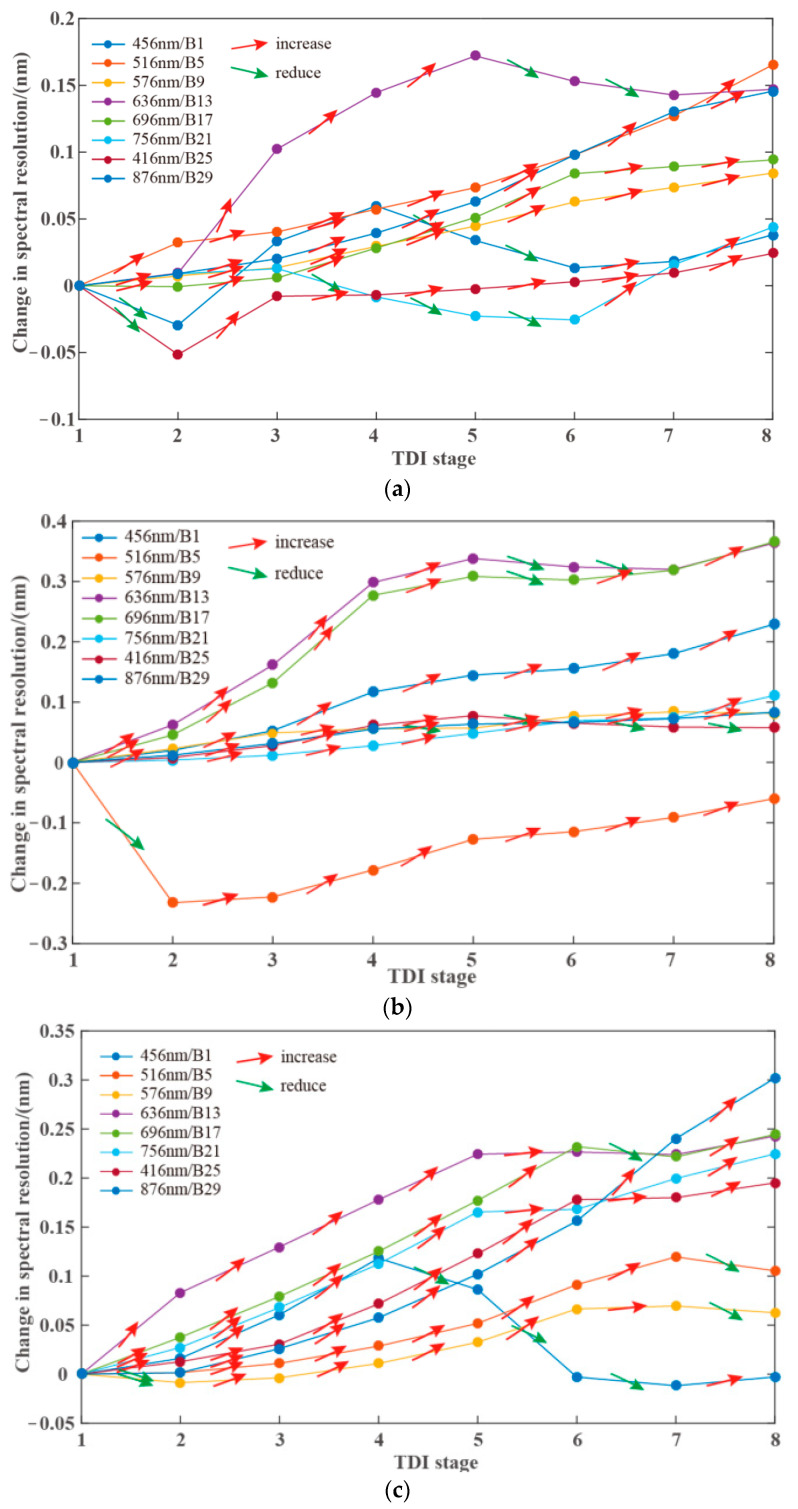
Spectral resolution of different integration stages of typical spectrum. (**a**) CMOS 1; (**b**) CMOS 2; (**c**) CMOS 3.

As mentioned in the second section, digital domain TDI uses successive rows of the detector as the same output spectral band, which may affect the spectral resolution of the hyperspectral camera. The effect of the integral level on the spectral resolution of the camera can be quantitatively analyzed by analyzing the response wavelength of the detector rows participating in digital domain TDI. We analyzed the spectral resolution of the three detectors at 8 output spectral bands, and gave the changes of their spectral resolution with the integral level, as shown in Figure 8.

We noticed that the integral level does affect the spectral resolution of the output spectrum, and as the integral level increases, the spectral resolution tends to increase mostly. It should also be noted that the increase in spectral resolution between different bands is different, and there is a case where the spectral resolution decreases with the increase of the integral level. The reason for these phenomena may be related to the measurement accuracy and the data fitting accuracy, or it may be related to the manufacturing process of the filter, but in any case, the variation of the spectral resolution of each band in the Figure 8 is within 0.4 nm. Therefore, we believe that when the integral level does not exceed level 8, the digital domain TDI has little influence on the spectral resolution.

## 4. In-Flight Test

**Figure 9:**
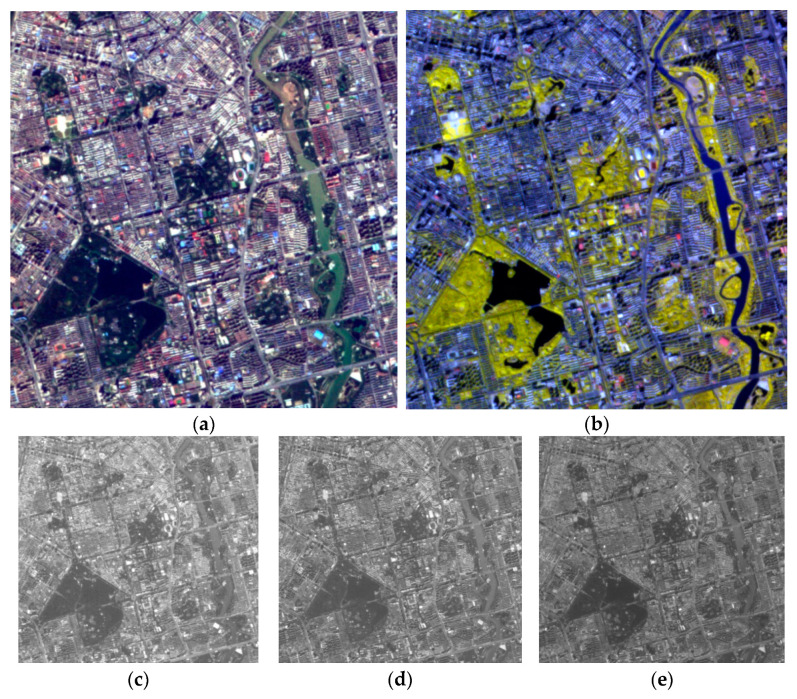

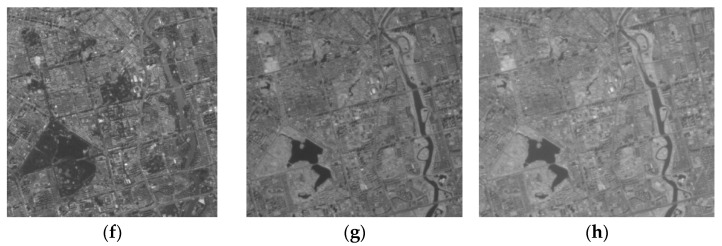
The image of Changchun City in Jilin Province captured in orbit. (**a**) The true color image composed of 486 nm, 548 nm, and 668 nm; (**b**) the false color image composed of 686 nm, 760 nm, and 835 nm; (**c**) the image of 486 nm; (**d**) the image of 548 nm; (**e**) the image of 668 nm; (**f**) the image of 686 nm; (**g**) the image of 760 nm; (**h**) the image of 835 nm.

After successfully launched into space, the hyperspectral camera executed a large number of imaging test instructions. The test results show that the camera is in a stable working state and can obtain hyperspectral images with a spatial resolution of 10 m and swath width of 150 km on a 500 km orbit. Moreover, the camera can adapt to different lighting conditions by changing the integral level of TDI in the digital domain. The image taken by the hyperspectral camera when it is overhead Changchun City, Jilin Province is shown in Figure 9. Figure 9a is the true color image of Changchun City composed of 486 nm, 548 nm and 668 nm, and Figure 9b is the corresponding false color image composed of 686 nm, 760 nm, and 835 nm. It is worth mentioning that the roads and houses can be clearly distinguished in Figure 9 and the image has a high signal-to-noise ratio. The spatial resolution shown in the image is conducive to the application of hyperspectral data in urban areas, and such signal-to-noise ratio can prove the effectiveness of digital domain TDI.

Compared with a small prism or grating hyperspectral camera with a ground resolution around 30 m, the hyperspectral camera we designed is weak in the number of spectral bands and spectral resolution, but it has a better performance in areas that require higher ground resolution. On the other hand, according to the analysis of the acquired hyperspectral images, the hyperspectral camera performs well in fields such as crop planting area statistics and large-scale water quality assessment, monitoring the water quality of the canal, and urban land planning. It can be said that the hyperspectral camera we designed has a good complementarity with small prism and grating hyperspectral cameras.

The camera can obtain relatively cheap hyperspectral images with high spatial resolution in a wide range, which means that it can quickly update high-quality hyperspectral data on the Earth’s surface and can contribute to the construction of a digital earth.

## 5. Conclusions

A light-compact and wide-coverage hyperspectral camera has been designed using a combination of linear variable filter splitting technology and digital domain TDI technology. The camera uses three linear variable filters close to the detectors to split light and it can obtain an ultra-wide image with more than 15,000 lines of pixels. The digital domain TDI technology discussed in the article had a good performance. On the one hand, it can equivalently increase the light throughput. The F number of the camera is 4.5, and the digital domain TDI used by the camera is level 8. Using the same parameters in Section 3.2, the SNR of the hyperspectral camera in the article is comparable to a camera with F number of 1.7. On the other hand, TDI has little influence on spectral resolution. The difference of spectral resolution caused by the digital domain TDI is less than 0.4 nm. During the hyperspectral camera working in orbits, we have obtained high-SNR images with a GSD of 10 m @ 500 km. Those images have shown great application potential in the areas where satellite hyperspectral images with high resolution are required. Therefore, the hyperspectral camera can make an important contribution to the construction of the digital earth.

## Figures and Tables

**Table 1:** The parameters of the hyperspectral camera.

Parameters	Value
Wavelength/nm	450–950
Spatial resolution@500 km/m	10
F number	4.5
Swath width/km	150
Pixel size/μm	4.25
Number of active pixels	5056 × 2968
Size of linear variable filter/mm	33.2 × 33.2 × 1
Effective area of linear variable filter/mm	21.5 × 12.7

**Table 2:** Signal-noise ratio analysis results (unit: dB).

Spectral Segment/nm	Spectral Resolution/nm	Irradiance/(W/m^2^)	Transmittance			*η* *·ε*	Integral Number			
			Lens Group	Filter	Optical System		1	2	4	8
B1/466	9	17.76	70.6%	43%	30.36%	69.63%	23.26	26.31	29.34	32.36
B7/556	11	21.1	75.6%	56%	42.34%	67.56%	26.13	29.16	32.18	35.20
B13/646	7.8	13.17	75.4%	64%	48.26%	69.73%	25.43	28.46	31.49	34.50
B20/751	9.1	12.47	76.1%	70%	53.27%	55.57%	25.29	28.46	31.35	34.36
B27/856	10.7	11.58	77.1%	76%	58.60%	33.03%	23.67	26.71	29.75	32.76
B32/931	11.6	10.54	77.2%	82%	63.30%	12.61%	19.69	22.78	25.83	28.86

## Data Availability

The data presented in this study are available on request from the corresponding author. The data are not publicly available due to the data involves business information.
